# Novel *DMD* Frameshift Variant (p.Leu2017Profs*5) in Spectrin‐Like Repeat 16 Expands the Mutational Spectrum of DMD

**DOI:** 10.1002/mgg3.70257

**Published:** 2026-07-01

**Authors:** Yu‐Chin Lin, G. W. Gant Luxton, Hwei‐Jen Lee, Yu‐Yang Lu, Hung‐Chi Yang, Kuo‐Sheng Hung, Ming‐Tsong Lai, Chih‐Fen Hu

**Affiliations:** ^1^ Department of Pediatrics, Tri‐Service General Hospital National Defense Medical University Taipei Taiwan; ^2^ Department of Molecular and Cellular Biology University of California Davis California USA; ^3^ Department of Biochemistry National Defense Medical University Taipei Taiwan; ^4^ Department of Medical Laboratory Science and Biotechnology Yuanpei University of Medical Technology Hsinchu Taiwan; ^5^ Center for Precision Medicine and Genomics, Tri‐Service General Hospital National Defense Medical University Taipei Taiwan; ^6^ KimForest Enterprise Co., Ltd. New Taipei City Taiwan; ^7^ Department of Pediatrics, School of Medicine, College of Medicine National Defense Medical University Taipei Taiwan

**Keywords:** AlphaFold structural modeling, computational variant interpretation, *DMD* gene, Duchenne muscular dystrophy, frameshift variant, structural analysis, whole‐exome sequencing

## Abstract

**Background:**

Duchenne muscular dystrophy (DMD) is an X‐linked neuromuscular disorder caused by pathogenic variants in the *DMD* gene, which encodes dystrophin, a cytoskeletal protein linking intracellular actin to the extracellular matrix via the dystrophin‐associated protein complex and maintaining muscle fiber integrity. With the emergence of disease‐modifying therapies, early and accurate molecular diagnosis is increasingly important. Whole‐exome sequencing (WES) is widely used to evaluate unexplained hyperCKemia and distinguish DMD from other inherited neuromuscular disorders.

**Results:**

WES identified a novel hemizygous frameshift variant in *DMD* (NM_004006.3:c.6050_6051del; p.Leu2017Profs*5) in a Taiwanese boy with markedly elevated creatine kinase levels (> 10,000 U/L) and clinical features consistent with DMD. The variant was classified as likely pathogenic according to ACMG criteria (PVS1, PS2, PP3). Comparative genomic analysis demonstrated strong evolutionary conservation at the variant site within the 16th spectrin‐like repeat (exon 42), with phastCons scores of 1 and phyloP scores of +2.925 and +1.015. AlphaFold‐based structural modeling suggested disruption of the three‐helix bundle architecture, while CHARMM‐based energy analysis suggested a potential destabilizing effect that appeared more consistent with previously reported pathogenic exon 42 frameshift variants than with benign missense variants at the same locus.

**Conclusions:**

This is the first reported case of DMD associated with the novel frameshift variant c.6050_6051del (p.Leu2017Profs*5). Integrated genomic, evolutionary, and structural analyses support the likely pathogenic interpretation of this variant. This study expands the mutational spectrum of DMD and highlights the value of combining WES with structure‐informed approaches for variant interpretation. These findings provide preliminary structural insights into the potential effects of the identified variant and highlight the possible utility of structure‐informed variant interpretation in the absence of functional assays.

## Graphical abstract

A novel DMD frameshift variant (c.6050_6051del; p.Leu2017Profs*5) causes a 2‐bp deletion in exon 42, leading to frameshift, premature truncation, and disruption of spectrin‐like repeat 16 architecture.

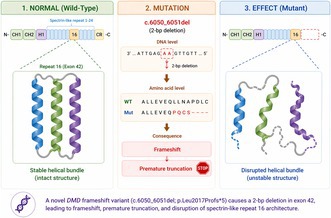

## 1 Introduction

Duchenne muscular dystrophy (DMD; OMIM #310200) is an X‐linked neuromuscular disorder characterized by progressive muscle weakness and marked hyperCKemia. It is caused by mutations in the *DMD* gene, which encodes dystrophin, a large cytoskeletal protein that maintains muscle fiber integrity by linking intracellular actin to the extracellular matrix through the dystrophin‐associated protein complex (DAPC), thereby providing mechanical stability during muscle contraction (Mercuri and Muntoni 2013; Birnkrant et al. 2018). Due to its large size, the *DMD* gene exhibits a high degree of allelic heterogeneity. Deletions represent the most frequent pathogenic variants, accounting for approximately 60%–70% of cases, followed by point mutations or small nucleotide substitutions (~20%–25%), duplications (~10%–15%), and other rearrangements such as inversions or insertions (~2%) (Aartsma‐Rus et al. 2016; Duan et al. 2021). These alterations disrupt the dystrophin reading frame in multiple ways, potentially resulting in transcripts subject to nonsense‐mediated decay, truncated unstable proteins, or variants with reduced functional capacity (Mercuri and Muntoni 2013; Aartsma‐Rus et al. 2016; Darras et al. 2000).

Although many *DMD* deletions have been catalogued in public databases, newly identified variants are often absent from these resources. Consequently, the clinical interpretation of novel variants in symptomatic individuals remains challenging when based solely on clinical features. Functional validation studies are frequently impractical in single‐case settings due to limited patient material, ethical considerations, and the time and cost required to establish disease‐relevant experimental models (Richards et al. 2015). Therefore, complementary computational approaches are increasingly used to support variant interpretation. Frameshift mutations may lead to alterations in protein conformation (Aartsma‐Rus et al. 2006), which can be modeled using AlphaFold (Jumper et al. 2021). In addition, in silico free‐energy calculations can be applied to evaluate the potential impact of such variants on protein structural stability, providing supportive evidence that may assist pathogenic variant interpretation. Comparison with previously reported pathogenic and benign variants further strengthens these interpretations (Sharma et al. 2014). These approaches enable structure‐informed interpretation of variants when functional validation is not feasible.

In this study, we describe a sporadic 6‐year‐old boy harboring a novel hemizygous frameshift deletion (c.6050_6051del; p.Leu2017Profs*5) in exon 42 of the *DMD* gene identified by whole‐exome sequencing (WES). This variant is absent from ClinVar (accessed March 2026) and classified as likely pathogenic according to ACMG/AMP guidelines. Through integrated clinical and structure‐based analyses, we provide supportive evidence for its pathogenicity. This study expands the mutational spectrum of DMD and demonstrates the utility of combining genomic and structural approaches for variant interpretation.

## 2 Materials and Methods

### 2.1 Human Subjects

This study included three individuals from a non‐consanguineous Taiwanese family, comprising the proband and his parents. Written informed consent was obtained from all participants or their legal guardians. The study was approved by the Institutional Review Board of Tri‐Service General Hospital (IRB No. C202615066). Clinical information was obtained from medical records and treating physicians.

### 2.2 Genomic DNA Extraction

Genomic DNA was extracted from peripheral blood leukocytes using a QIAamp Blood Mini Kit (Qiagen, Valencia, CA, USA) following the manufacturer's instructions.

### 2.3 Whole‐Exome Sequencing, Sanger Sequencing, and Variant Interpretation

WES of the proband was performed by KimForest Enterprise Co. Ltd. (New Taipei City, Taiwan), and the results were interpreted by a clinical genetics specialist (see author affiliations). Sequencing reads were aligned to the GRCh38/hg38 reference genome using the Illumina DRAGEN (version 4.2.6) end‐to‐end variant calling pipeline. Variants were filtered based on allele frequency, predicted functional impact, and clinical relevance. A hemizygous frameshift variant in the *DMD* gene (NM_004006.3:c.6050_6051del; p.Leu2017Profs*5) was identified.

Sanger sequencing of the proband and his parents was performed to determine whether the variant was inherited or occurred de novo. The coding region encompassing the variant site was amplified by polymerase chain reaction (PCR) using the following primers: forward, CCGTTTTACTAGACTTACCATC; reverse, GCCAACCACACTATCAAGTATT (product size: 502 bp). PCR products were purified using ExoSAP‐IT Express PCR Product Cleanup (Applied Biosystems) and subjected to Sanger sequencing (KimForest Enterprise Co. Ltd.).

Variant interpretation was performed using multiple databases, including VarSome (Kopanos et al. 2019), UniProt (UniProt Consortium 2023), ClinVar (Landrum et al. 2018), ClinVar Miner (Henrie et al. 2018), and dbSNP (Sherry et al. 2001). Allele frequency was assessed using the Taiwan BioBank and the Genome Aggregation Database (gnomAD). Variant classification was performed according to ACMG/AMP guidelines using VarSome and manual curation.

### 2.4 Comparative Genomics Analysis

To characterize the deletion at nucleotide resolution, the genomic region spanning *DMD* exon 42, including the c.6050_6051del site, was visualized using the Integrative Genomics Viewer (IGV) (Robinson et al. 2011). To assess functional relevance, evolutionary conservation was evaluated using cross‐species comparative genomics. The region was examined using the UCSC Genome Browser 100‐vertebrate alignment track, enabling sequence comparison across multiple vertebrate species.

Evolutionary conservation at the deletion site was quantified using two metrics: phyloP (Pollard et al. 2010), which measures nucleotide‐level conservation across a phylogenetic tree, and phastCons (Siepel et al. 2005), which estimates the probability that a nucleotide belongs to a conserved genomic element. These metrics provide complementary measures of evolutionary constraint.

### 2.5 Structural Modeling and Superimposition

A structural model of human dystrophin was obtained from the AlphaFold Protein Structure Database (https://alphafold.ebi.ac.uk) (Varadi et al. 2022). The region spanning residues Val1401‐Leu2800 was used to generate the Leu2017Profs*5 mutant model by introducing a frameshift at position Leu2017 using the Build Mutants protocol in Biovia Discovery Studio 2024 (San Diego, CA, USA). This protocol replaces the specified residues with the mutant sequence, samples rotamer conformations from a backbone‐dependent library, and selects the lowest‐clash conformation. The resulting structure was further optimized using the Smart Minimization algorithm to obtain an energetically favorable model. Structural superimposition of wild‐type and mutant models was performed for comparative analysis.

### 2.6 Free‐Energy Calculations to Assess the Effect of Mutations on Dystrophin Structural Stability

The potential energy of the wild‐type and Leu2017Profs*5 mutant dystrophin models was calculated using the Calculate Energy protocol in Biovia Discovery Studio 2024 (San Diego, CA, USA). This approach estimates the total potential energy of the system by summing bonded (bonds, angles, dihedrals, and impropers) and non‐bonded (van der Waals and electrostatic) interactions, providing a relative measure of structural stability. All calculations were performed using the CHARMM force field (Huang and MacKerell Jr. 2013).

For comparative analysis, additional exon 42 variants of the *DMD* gene were selected from the Leiden Open Variation Database (LOVD), including known pathogenic frameshift variants (p.Glu2003Asnfs*19 and p.Glu2013Ilefs*11) and likely benign missense variants (p.Leu2017Ile and p.Asn2019Ser). All variants were analyzed using the same computational protocol to compare their relative effects on protein stability. These calculations provide relative (rather than absolute) estimates of structural stability.

## 3 Results

### 3.1 Clinical Course of the Proband

The proband is a 6‐year‐old boy with a history of unexplained hypotonia, mild gross motor delay, attention‐deficit/hyperactivity disorder (ADHD), and autistic traits, who had been receiving rehabilitation therapy. He initially presented to the emergency department with abdominal discomfort and intermittent chest pain. On presentation, laboratory studies revealed markedly elevated creatine kinase (CK) levels (19,061 U/L), with normal cardiac markers. Liver enzymes (AST: 265 U/L, ALT: 569 U/L) and lactate dehydrogenase (LDH: 1164 U/L) were elevated. Urinalysis and renal function were normal. Initial evaluations showed no evidence of cardiac or acute systemic pathology. In the absence of dark urine or excessive exercise, rhabdomyolysis was considered less likely.

Neurological examination revealed a positive Gowers' sign, mild lumbar lordosis, and a waddling, wide‐based gait. The patient was able to stand on each foot individually but was unable to hop. No significant limb deformities were observed. Nerve conduction studies were normal, and electromyography was not performed due to tactile hypersensitivity. Based on these findings, a neuromuscular disorder was suspected, and genetic testing was performed.

The patient was discharged in stable condition with persistently elevated CK levels (7736 U/L). Oral prednisolone therapy was initiated for suspected DMD. At one‐month follow‐up, CK levels remained elevated (8440 U/L), and the North Star Ambulatory Assessment (NSAA) score was 26/34 (Scott et al. 2012), indicating preserved ambulation with emerging limitations in high‐demand motor tasks.

### 3.2 WES and Sanger Sequencing Identify a Likely Pathogenic Variant in *DMD*

**FIGURE 1:**
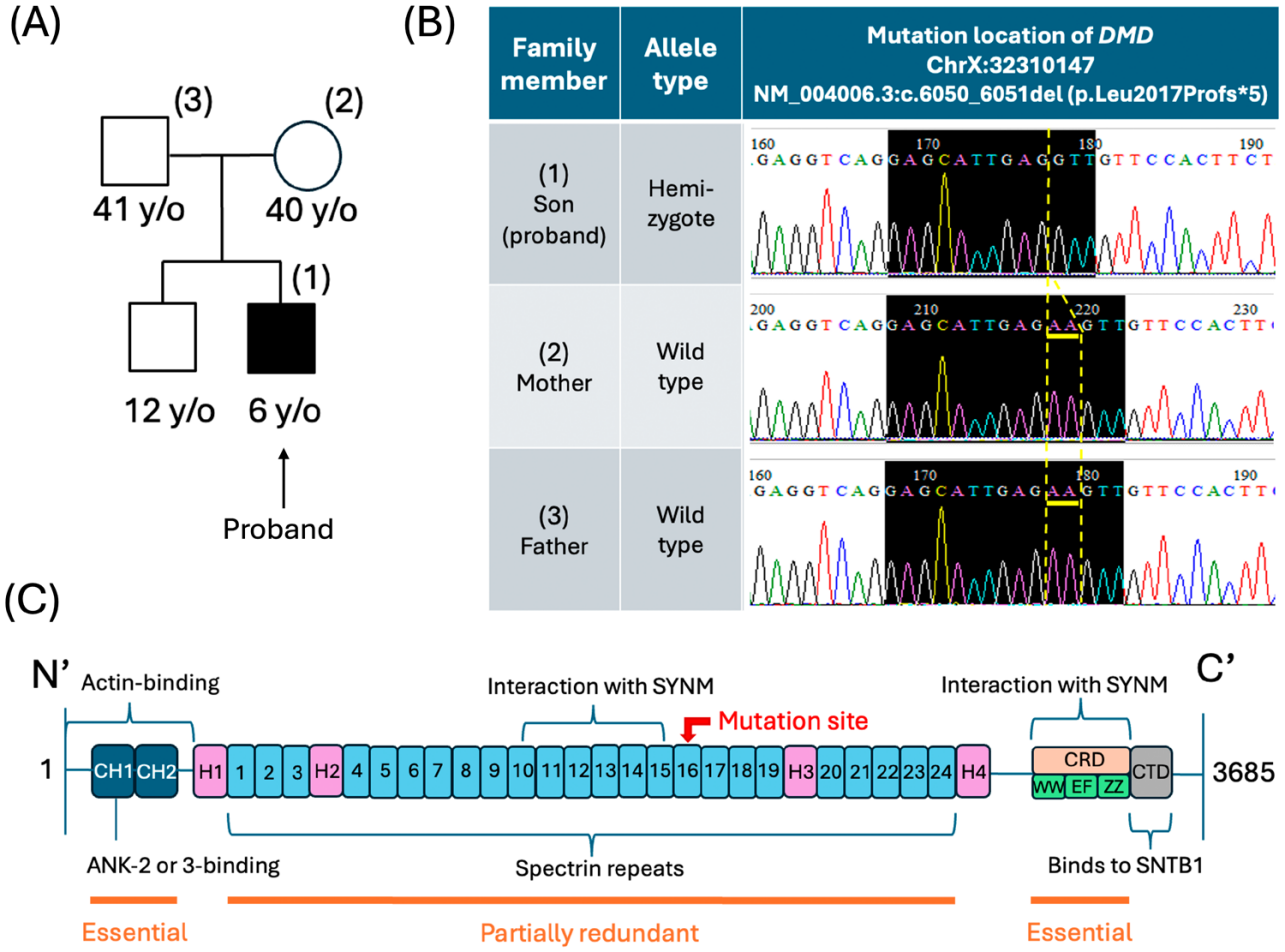
Family pedigree, Sanger sequencing, and localization of the *DMD* variant. (A) Pedigree of the family across two generations. The proband (black arrow) carries a de novo variant not detected in the maternal lineage. (B) Sanger sequencing confirms a two‐nucleotide (AA) deletion in the proband (*DMD* NM_004006.3:C.6050_6051del, indicated by the yellow dashed line) compared with his parents. (C) The variant c.6050_6051del (p.Leu2017Profs*5, red arrow) is located in exon 42 within the 16th spectrin‐like repeat of dystrophin, highlighting the structural context of the variant within the spectrin‐like repeat region. The N‐terminal and cysteine‐rich domains are essential for protein function, whereas parts of the central rod domain exhibit partial functional redundancy. CH, calponin homology; H, hinge; CRD, cysteine‐rich domain (including WW, EF‐hand, and ZZ domains); CTD, C‐terminal domain; SYNM, synemin; SNTB1, syntrophin beta 1; ANK, ankyrin.

WES, including mitochondrial DNA analysis, identified a novel hemizygous frameshift variant in the *DMD* gene (chrX:32,310,147; NM_004006.3:c.6050_6051del; p.Leu2017Profs*5). This variant introduces a premature termination codon and is predicted to result in loss of function, supporting PVS1‐level evidence (predicted loss‐of‐function) according to ACMG guidelines (Richards et al. 2015). Sanger sequencing confirmed that the variant occurred de novo in the proband and was not detected in his mother (Figure 1A,B), corresponding to PS2‐level evidence (confirmed de novo occurrence). The variant was absent from ClinVar, the Taiwan BioBank, and the Genome Aggregation Database (gnomAD). The variant is located in exon 42, which encodes the 16th spectrin‐like repeat within the central rod domain of dystrophin (Figure 1C). Based on ACMG/AMP criteria (PVS1, PS2), the variant was classified as likely pathogenic.

### 3.3 Evolutionary Conservation Analysis Supports the Functional Relevance of the Variant

**FIGURE 2:**
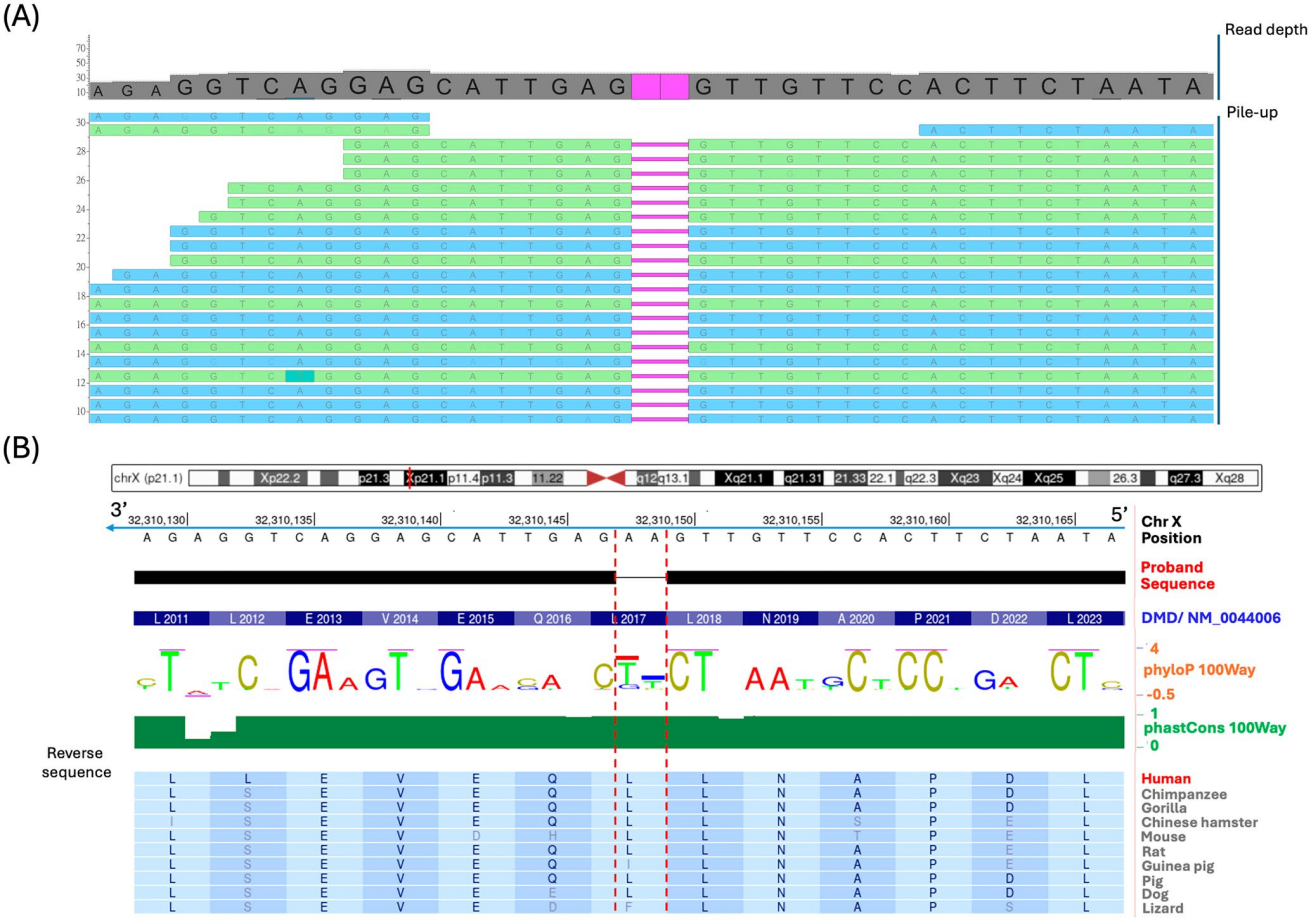
Comparative genomic analysis of the DMD exon 42 region showing the deletion site. (A) Integrative Genomics Viewer (IGV) screenshot showing a two‐nucleotide deletion (pink line) at chrX:32,310,147 in the proband, identified from high‐coverage next‐generation sequencing data. (B) Evolutionary conservation analysis of the genomic region using phyloP and phastCons. The variant c.6050_6051del (p.Leu2017Profs*5) is indicated by the red dashed line. The deletion site shows high conservation, with phastCons scores of 1 at both nucleotides and phyloP scores of 2.925 and 1.015. Multiple sequence alignment across nine vertebrate species, ranging from closely related (chimpanzee) to more distantly related species (lizard), suggests partial conservation of this region, indicating functional constraint at this locus. “100Way” denotes alignment across 100 vertebrate genomes.

To assess the functional relevance of the c.6050_6051del variant, evolutionary conservation analysis was performed for the genomic region encompassing exon 42 of the *DMD* gene, which encodes part of the 16th spectrin‐like repeat of dystrophin. Visualization of next‐generation sequencing data using IGV confirmed a two‐nucleotide deletion at chrX:32,310,147, with an average read coverage exceeding 30× (Figure 2A).

Cross‐species comparative analysis demonstrated strong evolutionary conservation at the variant site. The deleted nucleotides showed phastCons scores of 1 and phyloP scores of +2.925 and +1.015, indicating evolutionary constraint (Figure 2B). Multiple sequence alignment across nine vertebrate species, ranging from closely related (chimpanzee) to more distantly related species (lizard), suggests partial conservation of this region, indicating functional constraint at this locus. However, this pattern is not observed in avian species, and the absence of fish sequences in the UCSC Genome Browser limits comprehensive assessment across all vertebrate lineages. Together, the deleted nucleotides are located within an evolutionarily conserved region, providing limited computational observations that may partially support PP3 under ACMG guidelines (Richards et al. 2015).

### 3.4 Structural Modeling and Free‐Energy Calculations Suggest Destabilization of the Mutant Dystrophin

**FIGURE 3:**
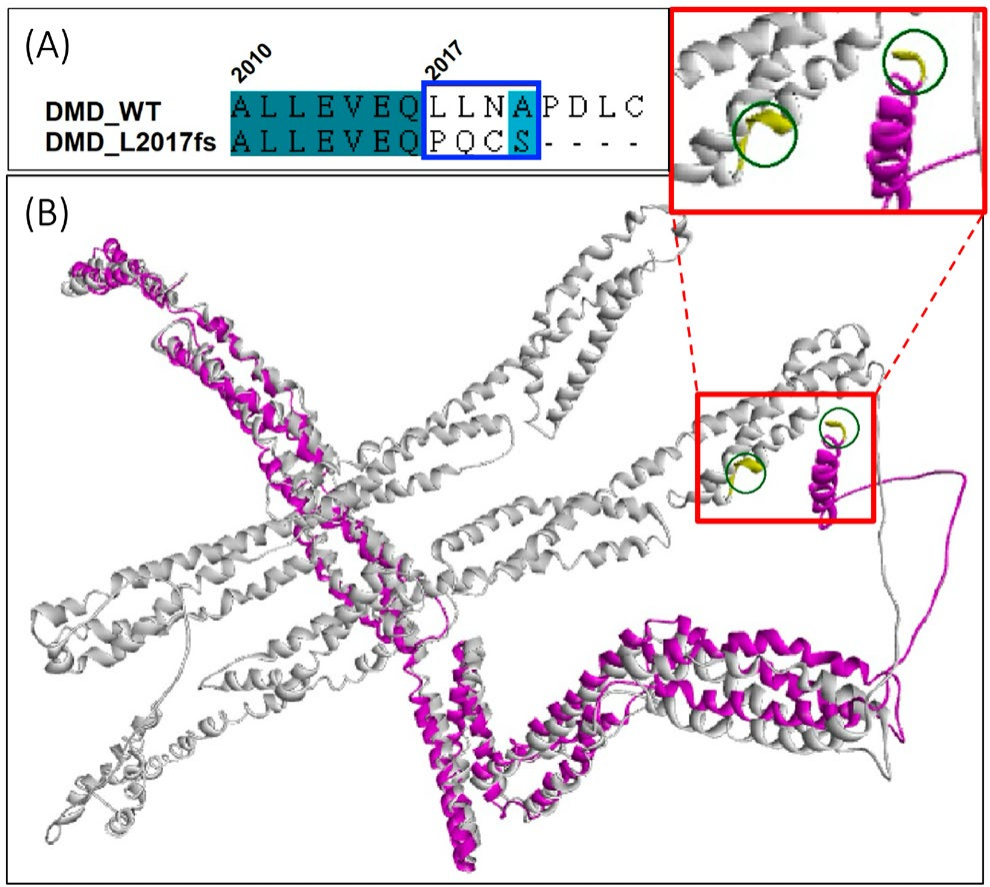
Predicted structural and sequence comparison of wild‐type and mutant dystrophin. (A) Sequence alignment of wild‐type and mutant dystrophin. The four terminal residues altered by the frameshift mutation are highlighted in blue boxes. (B) Structural superimposition of the wild‐type and mutant dystrophin models. The wild‐type model (residues Val1401‐Leu2800) was derived from the AlphaFold structure (AF_AFP11532F8). The mutant model is shown in magenta and the wild‐type model in grey. Green circles indicate the region containing the altered residues in both models. Notably, the mutant model appeared less able to maintain the characteristic three‐helix bundle architecture observed in the wild‐type structure, suggesting possible structural destabilization of the spectrin‐like repeat region.

To investigate the structural impact of the c.6050_6051del variant, AlphaFold‐based models of a large internal fragment of dystrophin (residues Val1401–Leu2800), encompassing the 16th spectrin‐like repeat, were analyzed for both wild‐type and mutant proteins. Sequence alignment revealed alterations at the C‐terminus, including residue changes and premature truncation in the mutant protein (Figure 3A).

Structural superimposition of the wild‐type and mutant models was performed using least‐squares fitting of Cα backbone atoms in Biovia Discovery Studio 2024. The overlaid structures revealed localized conformational differences, particularly in the region surrounding the variant site (Figure 3B). Notably, the mutant model appeared less able to maintain the characteristic helical bundle architecture of the spectrin‐like repeat observed in the wild‐type structure, suggesting disruption of the structural integrity of the spectrin‐like repeat domain.

**FIGURE 4:**
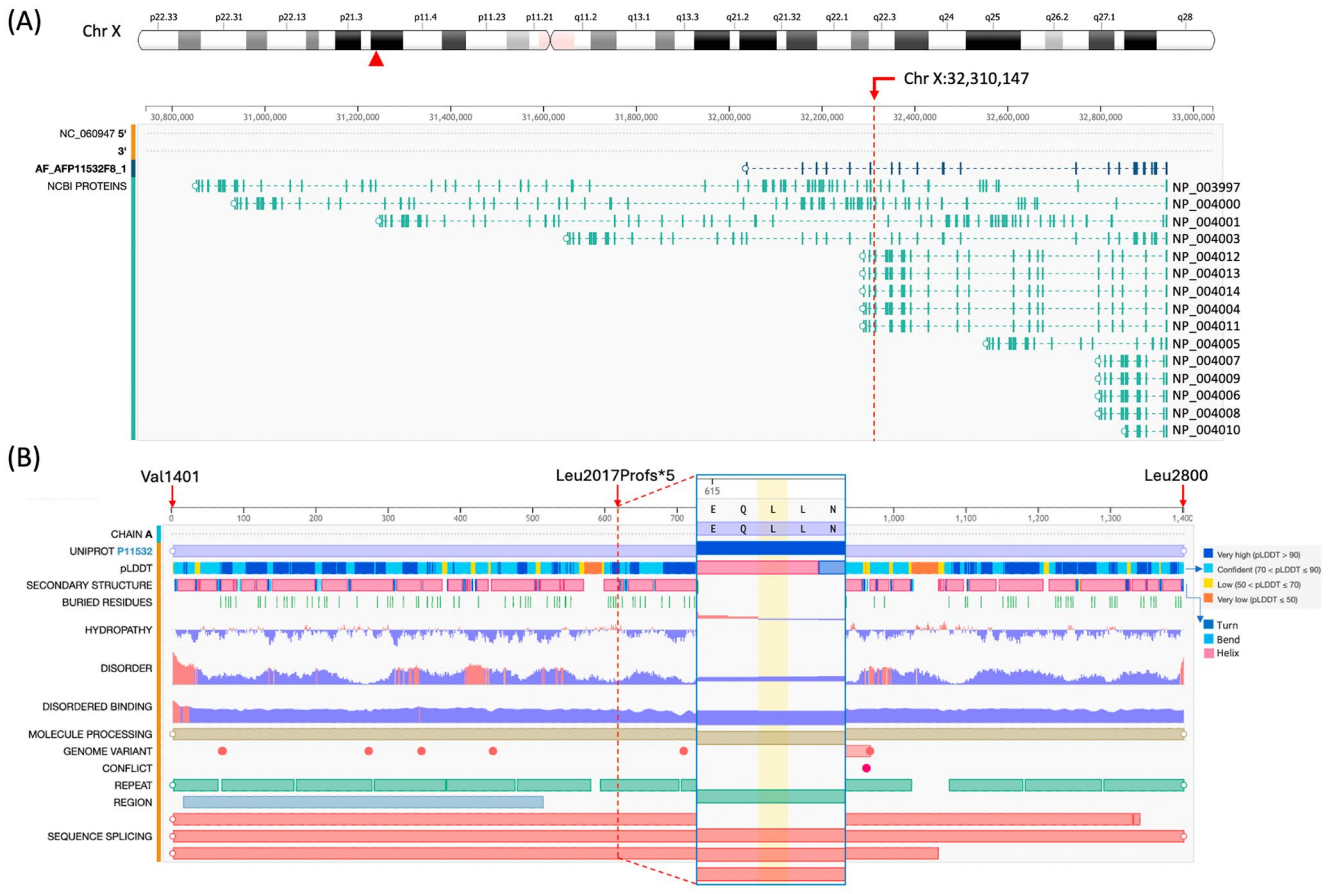
The c.6050_6051del frameshift affects multiple dystrophin isoforms and localizes to a structurally ordered, high‐confidence region. (A) Genomic mapping of the *DMD* locus (chrX:32,032,897–32,937,500) and dystrophin protein isoforms retrieved from the NCBI database. The mutation site (chrX:32,310,147; p.Leu2017Profs*5) is indicated by a red curved arrow and is present in nine isoforms, including NP_003997, NP_004000, NP_004001, NP_004003, NP_004012, NP_004043, NP_004014, NP_004004, and NP_004011, suggesting potential impact on major functional isoforms such as Dp427m. (B) Structural and sequence features of the dystrophin fragment (Val1401‐Leu2800), with the variant corresponding to residue 617 in the modeled structure. The variant site shows a high pLDDT score (92.52), indicating high confidence in the predicted structure. Intrinsic disorder prediction (IUPred2A) shows a low disorder probability (0.13), suggesting that this region is structurally ordered rather than intrinsically disordered. Together, these features support the functional relevance of the affected region and its potential impact on dystrophin structural stability. pLDDT, predicted local distance difference test.

To assess the broader impact of the variant, exon 42 was mapped across known dystrophin isoforms. Nine of fifteen protein isoforms retrieved from the NCBI database, including the predominant full‐length muscle isoform Dp427m, contain this region, suggesting that the variant may affect major functional isoforms relevant to skeletal muscle physiology (Figure 4A).

Structural confidence and regional properties were further evaluated. The predicted local distance difference test (pLDDT) score at the variant site was 92.52, indicating high confidence in the modeled structure. Intrinsic disorder prediction using IUPred2A (Mészáros et al. 2018) showed a low disorder probability (0.13), suggesting that this region is structurally ordered. Variants in such ordered regions may be more likely to influence protein stability and function (Figure 4B). Together, these findings support the structural and functional relevance of the affected region.

To assess structural impact, the potential energy of the wild‐type and mutant models was calculated using the CHARMM force field in Biovia Discovery Studio 2024. This approach estimates relative structural stability by summing bonded and non‐bonded energy terms. For comparison, additional exon 42 variants reported in the LOVD were analyzed using the same computational protocol. The Leu2017Profs*5 variant showed a pattern of relative destabilization similar to that observed in previously reported pathogenic frameshift variants (p.Glu2003Asnfs*19 and p.Glu2013Ilefs*11). In contrast, likely benign missense variants (p.Leu2017Ile and p.Asn2019Ser) showed minimal changes in energy relative to the wild‐type structure, consistent with their reported benign classification (Table 1).

**TABLE 1:** Comparative analysis of *DMD* (NM_004006.3) exon 42 variants including ACMG classification, conservation scores, and calculated energy values.

Genomic variant (chrX)		32,310,192 delCA	32,310,163 insTAAT	32,310,150 G>T	32,310,147 delAA	32,310,143 T>C
Transcript		c.6006_6007del	c.6033_6036dup	c.6049C>A	c.6050_6051del	c.6056A>G
Protein		p.Glu2003Asn fs*19	p.Glu2013Ile fs*11	p.Leu2017Ile	p.Leu2017Pro fs*5	p.Asn2019Ser
Varsome score		9	9	−7	9	−2
ACMG Class		Likely pathogenic	Likely pathogenic	Benign	Likely pathogenic	Likely benign
ACMG Criteria^a^		PVS1 PM2	PVS1 PM2	BS2 BP4 BP1	PVS1 PM2	BP4 BP1 PM2
Conservation score (Varsome)	PhastCons100way	1.000, 1.000	1.000, 0.993, 0.996, 0.517, 0.281, 0.998	1.000	1.000, 1.000	1.000
	PhyloP 100way	5.698, 0.746	5.698, 0.257, 3.565, 0.854, −0.525, 6.720	2.793	1.015, 2.925	2.453
Calculated energy of DMD structural model (kcal/mol)^b^		−43,721	−44,298	−99,616	−44,172	−100,045

^a^ PVS1: very strong, PM2: supporting, BS2: strong, BP4: moderate, BP1: supporting.

^b^ Calculated energy for wild‐type: −100,276 kcal/mol. The reported energy values represent relative computational estimates derived from in silico structural analyses and should not be interpreted as experimentally validated functional measurements.

Taken together, these computational analyses suggest that the Leu2017Profs*5 variant may alter the structural properties of the spectrin‐like repeat region of dystrophin (Harper et al. 2002).

## 4 Discussion

We report a 6‐year‐old boy in whom a novel hemizygous frameshift variant in the *DMD* gene was identified by WES, resolving a diagnostically challenging presentation complicated by co‐occurring ADHD and autistic traits. Beyond variant identification, this case provides clinically relevant insights into diagnostic strategy, phenotypic interpretation, and structure‐informed variant characterization.

First, this case highlights the diagnostic challenges of DMD prior to the implementation of newborn screening programs. Taiwan's Health Promotion Administration initiated CK‐MM–based dried blood spot screening in January 2022; however, this patient was born before its implementation and therefore did not benefit from early detection. In addition, CK‐MM–based screening strategies are not fully sensitive and may still result in delayed diagnosis in some patients (Zhang et al. 2025; Alsaedi 2025). This case therefore illustrates the complementary role of genomic testing, particularly in patients with atypical presentations and delayed recognition of neuromuscular symptoms. Moreover, precise nucleotide‐level characterization remains important for therapeutic decision‐making, as the c.6050_6051del variant identified in this patient is not amenable to currently approved exon‐skipping therapies.

Second, this case illustrates how co‐occurring neurodevelopmental conditions may obscure early manifestations of neuromuscular disease. Hypotonia and motor delays may coexist with autism spectrum disorder. In addition, executive dysfunction, sensory processing differences, and motor coordination deficits associated with autism spectrum disorder may reduce physical activity and potentially mask underlying muscle weakness. However, these findings should also prompt evaluation for underlying neuromuscular disorders when clinically indicated (Zhang et al. 2025; Alsaedi 2025). Similar motor abnormalities have also been reported in children with ADHD (Athanasiadou et al. 2020) and individuals with co‐occurring autism spectrum disorder and ADHD exhibit further reductions in motor competence (Dionisio et al. 2024). In this patient, the combination of neurodevelopmental comorbidities and an atypical presenting complaint of abdominal pain delayed recognition of underlying muscular dystrophy, with classical signs such as Gower's sign and waddling gait only recognized upon targeted neurological examination. These findings emphasize the importance of maintaining a low threshold for serum CK testing and genetic evaluation in children presenting with unexplained motor abnormalities, regardless of coexisting neurodevelopmental diagnoses. The proband's NSAA score of 26/34 indicates preserved ambulation with emerging deficits in high‐demand motor tasks, consistent with the early ambulatory phase of DMD, during which motor performance typically peaks before progressive decline (Zambon et al. 2022). This stage represents a critical therapeutic window. Corticosteroid treatment initiated during the early ambulatory phase has been shown to improve motor function trajectories and delay loss of ambulation (Ricotti et al. 2016). Therefore, timely recognition of DMD has direct implications for disease‐modifying management and long‐term functional outcomes.

From a molecular perspective, this study supports the utility of structure‐informed variant interpretation. Prior studies have demonstrated that molecular dynamics simulations can elucidate the structural and functional consequences of dystrophin variants, particularly within spectrin‐like repeat domains that contribute to the mechanical stability of the sarcolemma (Legrand et al. 2011; Fealey et al. 2018). The variant identified in this patient localizes to spectrin‐like repeat 16 within the central rod domain, a region that functions as an elastic spacer and mechanical buffer during muscle contraction. Our AlphaFold‐based structural modeling and free‐energy analysis suggest that the p.Leu2017Profs*5 frameshift disrupts the characteristic three‐helix bundle structure and suggests a degree of structural destabilization that may resemble patterns observed in previously reported pathogenic exon 42 variants. These findings may be consistent with the proposed role of the rod domain in maintaining sarcolemmal integrity under mechanical stress. However, these results should be interpreted with caution, as in silico structural predictions cannot fully capture the complex cellular and biomechanical context of dystrophin function. In addition, the reliability of AlphaFold predictions for frameshift‐truncated proteins remains incompletely validated, and static energy calculations may not fully reflect conformational dynamics in vivo. Comparisons between truncating and missense variants should therefore be interpreted cautiously. Furthermore, no muscle biopsy, dystrophin protein quantification, or western blot analyses were available for experimental validation of the predicted structural effects.

Finally, this case illustrates how evolutionary conservation and computational structural analyses may provide complementary supportive information for variant interpretation when functional studies are not immediately feasible. Nevertheless, such computational observations remain supportive under ACMG/AMP criteria and cannot substitute for experimental validation (Bean and Hegde 2017; Gunning and Wright 2023; Tsishyn et al. 2024). Future studies using patient‐derived myotubes or model systems to directly assess dystrophin expression and sarcolemmal stability would further clarify the functional impact of the p.Leu2017Profs*5 variant and may refine its classification.

Taken together, this study highlights the importance of integrating clinical evaluation, genomic diagnostics, and structure‐based approaches for the interpretation of novel *DMD* variants. It further illustrates how neurodevelopmental comorbidities may obscure early neuromuscular signs, underscoring the need for heightened clinical vigilance and early diagnostic testing in pediatric practice. This approach may be particularly valuable in clinical settings where functional validation is not readily feasible, providing an additional layer of evidence for variant classification.

## 5 Conclusion

Integrated genomic and computational analyses provided supportive evidence for the likely pathogenic interpretation and potential functional impact of this variant. This report expands the mutational spectrum of DMD and highlights the potential role of structure‐informed approaches as complementary tools for variant interpretation.

## Author Contributions

Y‐.C.L. and Y‐.Y.L., general pediatricians involved in the patient's primary care, contributed to clinical evaluation, data acquisition, interpretation of clinical findings, and manuscript drafting. C‐.F.H., a pediatric neurologist specializing in neuromuscular disorders, contributed to study conceptualization, neurological assessment, interpretation of neuromuscular findings, and manuscript preparation. K‐.S.H., M‐.T.L., and H‐.C.Y. contributed to the analysis and interpretation of genetic testing results, while M‐.T.L. additionally designed the primers for DMD variant confirmation and prepared the figures. H‐.J.L. performed computational structural modeling and stability analyses of the wild‐type and mutant dystrophin proteins. G.W.G.L. conducted critical mock review and contributed to scientific writing, logical organization, and refinement of the manuscript. Specifically, his critical scientific feedback led to further evaluation and strengthening of the study findings. He also contributed to the interpretation of the results and to the development of the key scientific arguments presented in Section 4. All authors have read and approved the final manuscript.

## Funding

This work was supported by Tri‐Service General Hospital (grant numbers: TSGH‐D‐114050 and TSGH‐D‐115060).

## Conflicts of Interest

The authors declare no conflicts of interest.

## Data Availability

The WES data generated in this study have been deposited in the NCBI Sequence Read Archive (SRA) under BioProject accession number PRJNA1449744 (https://www.ncbi.nlm.nih.gov/bioproject/PRJNA1449744). The AlphaFold model of DMD (AF_AFP11532F8) with the sequence from Val1401 to Leu2800 was used for the structural data analysis in the manuscript. The datasets generated and analyzed during the current study are available in the UniProt repository, UniProtKB accession P11532 (https://www.uniprot.org/uniprotkb/P11532).
